# Comparison of the midterm result between locking plate and elastic intramedullary nail treating oblique ulnar fracture Bado type I acute monteggia fracture in pediatric patients

**DOI:** 10.1186/s12891-022-05809-5

**Published:** 2022-09-12

**Authors:** Djandan Tadum Arthur Vithran, Zhenqi Song, Kongjian Wang, Zhongwen Tang, Feng Xiang, Jie Wen, Sheng Xiao

**Affiliations:** 1 Department of Pediatric Orthopedics, Hunan Provincial People’s Hospital, the First Affiliated Hospital of Hunan Normal University, Changsha, 410005 Hunan China; 2 Department of Human Anatomy, Hunan Normal University School of Medicine, Changsha, Hunan 410013 China

**Keywords:** Elastic intramedullary nail, Locking compression plate, Bado type I acute monteggia fracture, Oblique ulnar fracture

## Abstract

**Background:**

Acute Monteggia fracture (AMF) is defined as a fracture of the proximal ulna combined with a dislocation of the radial head, with less than 1 percent prevalence in pediatric patients with an elbow injury. This retrospective study aimed to compare the efficacy of two treatment methods for oblique ulnar fracture AMF Bado type I in children.

**Methods:**

In this retrospective study, 28 children with oblique ulnar fracture acute Monteggia injury Bado I were included. Patients was divided into two groups: In the EIN group 16 patients were fixed with elastic intramedullary nail after reduction, and in the LCP group 12 patients were fixed with locking compression plate after reduction. Operation time and blood loss were recorded and the patients were assessed clinically by the Mayo Elbow Performance Score (MEPS), range of movement(ROM) and range of rotation(ROR).

**Results:**

Twenty-eight patients were accessible for durable follow-up, with a mean age of 7.7 ± 1.3 years, follow up by 33.7 ± 5.1 months. The average operation time was 48.1 ± 9.2 min for the EIN group and 67.1 ± 7.2 min for the LCP group. The average blood loss was 8.2 ± 2.3 ml for the EIN group and 18.8 ± 3.8 ml for the LCP group. The average operation time and average blood loss in the EIN group are significant less than the LCP group. Mean MEPS, ROM, ROR in both group improved significantly before sugery to three months after surgery, and maintained very good results at last follow up. There is no significant difference between the EIN group and the LCP group in MEPS, ROM and ROR.

**Conclusions:**

Oblique ulnar fracture Bado type I acute monteggia fracture in pediatric patients fixed by EIN and LCP can both get good mid-term results measured by MEPS, ROM and ROR, Fixed by EIN have shorter operation time and less blood loss than fixed by LCP.

## Introduction

Monteggia fracture was first described by an Italian surgeon, Giovanni Batista Montage, in 1814 as an ulnar fracture, usually in the proximal edge with an associated radial head dislocation [1]. In 1967 Bado classified Monteggia fractures into four types according to the direction of radial head dislocation and ulnar shaft angulation [2]. Monteggia fracture is a rare injury usually seen in children between four to ten years old, with an incidence of less than 1% of all pediatric fractures [3]. It is generally agreed that these injuries should be managed in the acute state [4].

Treatment of Bado type I acute Monteggia fractures in children remains a challenge for pediatric orthopedic surgeons, and can vary from conservative treatment by cast to surgical treatment [5]. The critical feature of managing Monteggia fractures is to ensure the length stability of the reduced ulnar and radial head [6, 7]. When the length stability of ulnar cannot be maintained as oblique fracture, it is indicated to use surgical stabilization by elastic intramedullary nail (EIN) or locking compression plate (LCP) [8, 9]. Elastic intramedullary nail has lower rates of recurrent radial-head subluxation and loss of ulnar alignment requiring subsequent operative treatment [8]. But plate fixation offers excellent stability, fully early motion,allows to manage proximal and distal fractures, and does not need a cast in post-operative periods [10]. However, no specific research has compared these two procedures. Therefore, we compared the treatment methods employed for Bado type I acute Monteggia fractures to improve the understanding of the diagnosis and therapeutic approach of these fractures.

## Materials and methods

### Study population

Children with elbow fracture treated at our department between January 2013 and August 2020 were enrolled in this study. All subjects met the inclusion criteria:

- Inclusive criteria: 1. children aged below 14 years old; 2. diagnosed as Bado type I acute Monteggia fracture, 3.X-ray confirmed oblique ulnar fracture, and 4. children who were followed up for at least 2 years.
- Exclusion criteria: Open fracture or fracture associated with vascular injuries, incomplete, transverse and comminuted ulnar fractures.

Patients treating with EIN were admitted into the EIN Group and patients treating with LCP were admitted into the LCP Group. A total of 28 patients, comprising of 18males and 10 females, with an average age of 7.7 ± 1.3 years (6–10 years) were eligible. There were 16 cases of right elbow injury and 12 cases of left elbow injury; 19 cases suffered falling injury and 9 cases suffered road traffic injury.

### Surgical procedures

The patient was placed in the supine position and given general anesthesia.Tourniquet was used to reduce blood loss. In the EIN group, the ulna was first reestablished by longitudinal traction. After reduction of ulnar, a longitudinal incision was made about 1.5 cm long outside the proximal ulna to exposed the ulnar and a suitable diameter EIN was inserted inside ulna. In the LCP group, a longitudinal incision, centered at the apex of the fracture, was made along the subcutaneous border of the ulna, at the extensor carpi ulnaris–flexor carpi ulnaris interval. Thereafter, the ulnar fracture was reduced and fixed by suitable LCP. In both groups, after completing the reduction of ulnar, the elbow was flexed and posteriorly directed pressure over the anterior aspect of the radial head for reduction of the radial head, and the forearm was pronated and supinated to achieve a concentricreduction. Fluoroscopy confirmed that the ulna was aligned well, and the radial head was satisfactorily reducedwith the forearm in supination. Thereafter. a long arm splint or bivalved cast was applied with the elbow in 90—110 degrees of flexion andforearm supination for 4–6 weeks.

### Data collection

Surgical records were reviewed, and the operation time and blood loss were recorded. Mayo Elbow Performance Score (MEPS), range of elbow movement flexion/extension(ROM),and range of rotation(ROR) were recorded before the operation, three months postoperation and the last follow-up (Table 1). All MEPS were filled by patients along with their parents, and pre-operation MEPS were filled by the parents.

**Table 1:** General information of selected children

No	Age (yo)	Gender	Group	Side	FU (m)	OP (min)	Blood Loss (ml)	Pre-OP			3 M Post-OP			LFU Post-OP		
								MEPS	ROM	ROR	MEPS	ROM	ROR	MEPS	ROM	ROR
1	6	M	EIN	R	42	40	5	20	20	10	75	70	100	90	130	150
2	7	F	LCP	L	33	65	15	15	15	20	70	75	95	85	130	150
3	8	F	EIN	R	26	48	10	25	25	5	70	75	90	85	140	160
4	7	M	EIN	L	37	53	10	15	15	15	80	80	80	95	140	160
5	8	M	LCP	L	41	70	20	20	20	10	65	80	90	90	140	160
6	8	M	LCP	R	35	67	15	25	15	15	75	90	100	90	130	150
7	7	M	EIN	R	40	38	10	30	25	10	75	85	95	90	130	140
8	10	M	LCP	R	36	78	15	25	10	5	70	80	85	95	130	150
9	8	F	EIN	L	31	32	10	25	20	10	70	80	95	90	135	150
10	9	M	EIN	L	29	45	5	15	15	15	70	85	90	90	135	150
11	6	M	EIN	R	26	39	5	20	15	10	75	75	95	95	140	160
12	9	M	LCP	L	28	80	25	20	15	10	75	75	90	90	130	160
13	7	F	EIN	R	41	61	10	15	10	5	70	70	100	90	130	140
14	6	M	EIN	L	40	55	6	15	15	10	80	80	100	85	140	150
15	8	M	EIN	R	32	44	7	20	20	15	80	90	105	90	140	150
16	6	F	LCP	R	32	65	20	15	10	5	70	70	80	85	135	140
17	8	M	LCP	R	31	70	20	15	20	15	75	75	85	90	140	135
18	8	M	EIN	R	27	48	8	25	20	10	75	85	100	95	135	160
19	7	F	LCP	R	38	55	15	20	15	10	80	70	100	85	130	150
20	9	M	EIN	L	39	50	10	15	15	10	85	90	95	90	140	150
21	6	M	LCP	L	27	70	20	15	15	15	80	75	90	85	130	140
22	10	F	EIN	R	35	50	10	10	5	5	70	70	80	85	120	130
23	6	M	EIN	R	35	41	5	20	10	10	75	75	90	90	135	140
24	9	M	LCP	L	30	60	15	20	20	10	75	80	105	90	140	150
25	7	F	EIN	L	30	65	10	25	20	15	75	80	100	95	140	150
26	10	F	LCP	R	29	65	25	25	10	10	75	70	85	90	130	150
27	7	M	LCP	L	32	60	20	20	15	20	80	75	85	90	135	140
28	9	F	EIN	R	40	60	10	15	10	10	70	85	100	90	130	160

### Statistical analysis

SPSS 21.0 was used for statistical analysis. The operation time, bloodloss, MEPS score,ROM and ROR were expressed as the mean ± SD. All measured data pre-operation and three months postoperation and the last follow-up were compared using the Mann–Whitney rank sum test. A *p* < 0.05 was considered as statistically significant.

## Results

All 28 patients sustained an oblique ulnar fracture. All cases were followed-up for33.7 ± 5.1 months (Table 1). The operation time was 48.1 ± 9.2 min for the EIN Group and 67.1 ± 7.2 minfor the LCP Group. The bloodloss was 8.2 ± 2.3 ml for the EIN Group and 18.8 ± 3.8 ml for the LCP Group. The operation time and bloodloss in the EIN Group were significant less than the LCP Group (*p* < 0.05). MEPS in the EIN Group improved from 19.4 ± 7.2 before surgery to 74.7 ± 4.6 at three months after surgery, and maintained 90.3 ± 3.4 at the last follow up. ROM in the EIN Group improved from 16.3 ± 5.6 before surgery to 79.7 ± 6.7 at three months after surgery, and maintained 135.0 ± 5.8 at the last follow up. ROR in the EIN Group improved from 10.3 ± 3.4 before surgery to 94.7 ± 7.2 at three months after surgery, and maintained 150.0 ± 8.9 at the last follow up. There was a significant difference among the pre-operation, three months post-operation and the last follow-up scores in MEPS, ROM and ROR (*p* < 0.05). MEPS in the LCP Group improved from 19.6 ± 4.0 before surgery to 74.2 ± 4.7 at three months after surgery, and maintained 88.8 ± 3.1 at the last follow-up (*p* < 0.05). ROM in the LCP Group improved from 12.1 ± 5.0 before surgery to 76.3 ± 5.7 at three months after surgery, and maintained 133.3 ± 4.4 at the last follow-up. ROR in the LCP Group improved from 10.3 ± 3.4 before surgery to 90.8 ± 7.6 at three months after surgery, and maintained 147.9 ± 7.8 at the last follow up. There was a significant difference among the preoperation, three months post-operation and the last follow-up scores in MEPS, ROM and ROR (*p* < 0.05). However, there was no significant difference in MEPS, ROM and ROR between the EIN Group and the LCP Group no matter before the operation, three months postoperation and the last follow-up (*p* > 0.05).

## Discussion

Bado classification of Monteggia fractures has been proved a exellent classification with only minimal modifications except for the addition of various equivalent lesions [11]. Bado type I refers to anterior dislocation of the radial head associated with an apex anterior ulnar diaphyseal fracture at any level, which is the most common type of Monteggia fracture (60 -79%) [12, 13].

For Bado type IMonteggia fracture, non-surgical therapy should be considered in case of length stable ulnar fracture just like greenstick [14]. Numerous researchers reported that closed reduction associated with casting is one of the most effective treatment methods for patients with acute Monteggia fractures, which typically result in unpredictable healing [15, 16]. Numerous studies investigated the therapeutic techniques and recommendations for acute Monteggia fractures, which is a non-invasive treatment with a cast focused on stabilizing ulnar fractures [8, 17, 18]. Ring recommended a treatment approach to reconstruct ulnar anatomy to support acute Monteggia fracture displacements, stating that incomplete ulnar fractures, such as plastic deformation and greenstick fractures, are usually treated by closed reduction with casting, complete fractures should be treated with surgical reduction and stabilization [19]. Intramedullary pin fixation is typically advised for stable length ulnar fractures, such as short oblique or transverse fractures. Open reduction and plate fixation are advised for unstable length ulnar fractures, such as comminuted or long oblique fractures, but there is no clear definition of short or long oblique fractures [20].

Several trials have demonstrated that elastic stable intramedullary nailing (ESIN) is a minimally invasive and effective procedure with a low risk of complication, which can be used in children with acute Monteggia fractures needing surgical care as an initial treatment choice [21]. However, the most appropriate therapeutic approach for acute Monteggia fractures remains controversial [22, 23]. There is no consensus on the optimal surgical option to provide better outcome for Bado type I Monteggia fracture with oblique ulnar fracture.

**Fig. 1:**
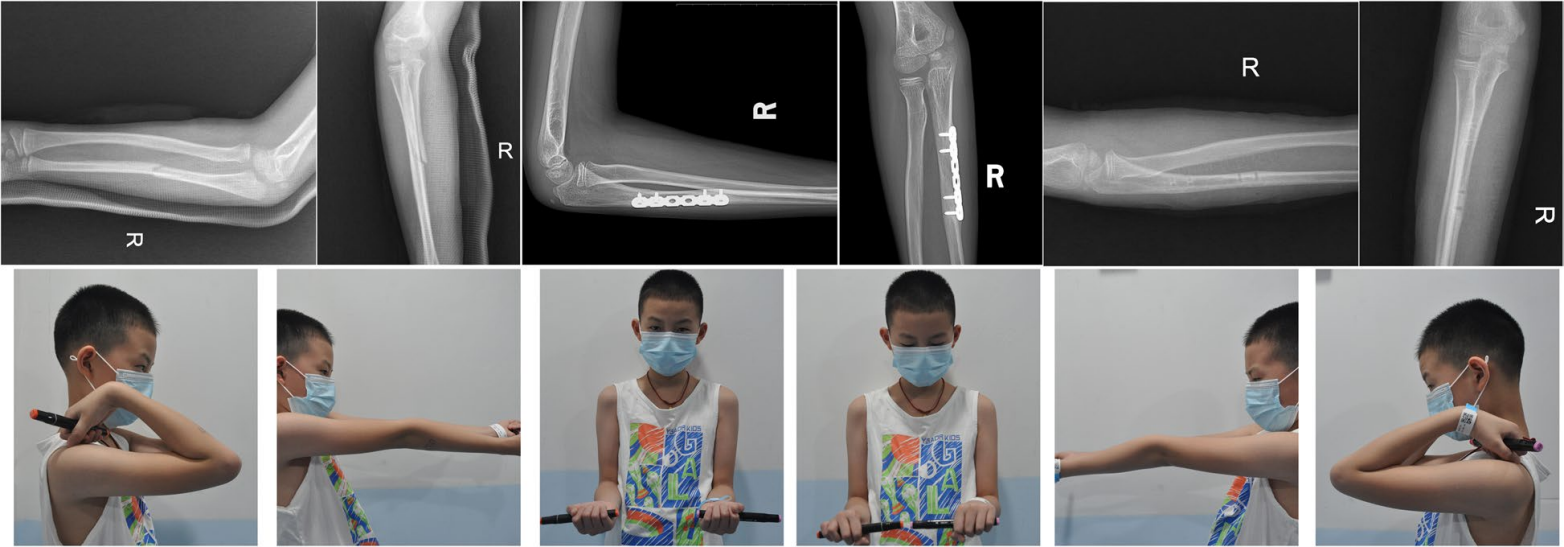
Case No.17, 8 years old boy, diagnosed as oblique ulnar fracture AMF Bado type I, treated with locking plate, after 31 months FU, the movement of elbow return normal

**Fig. 2:**
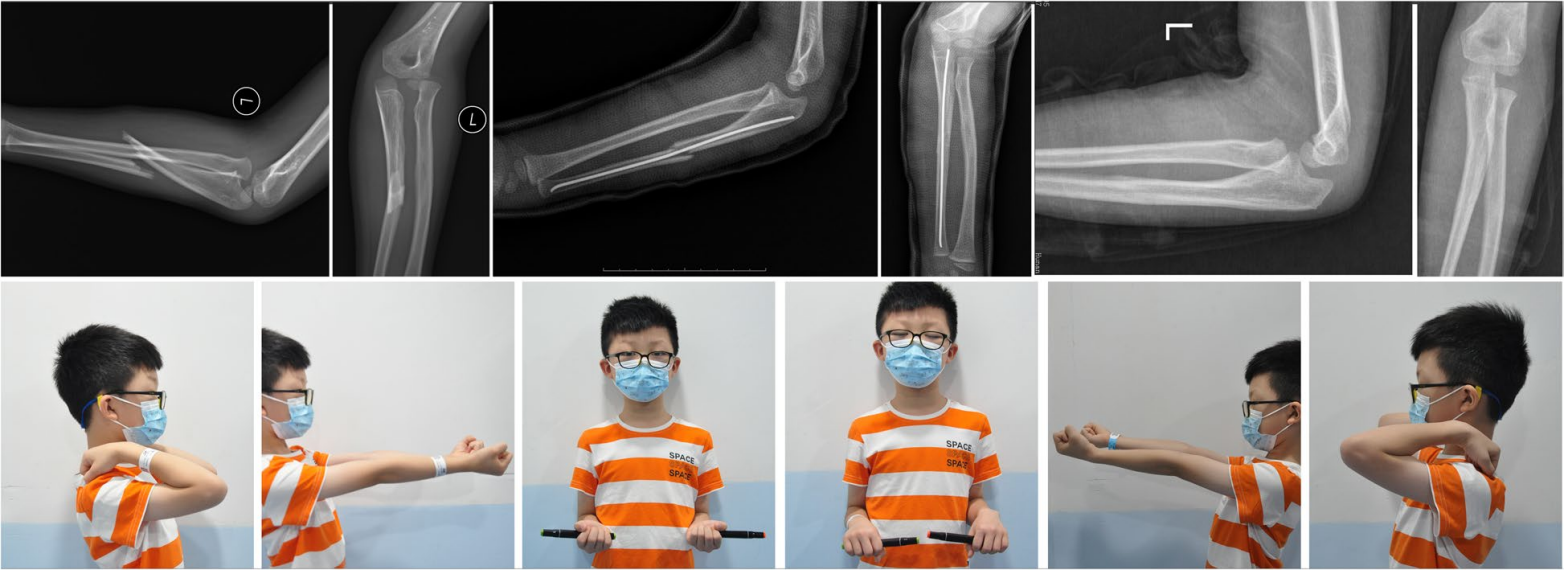
Case No.4, 7 years old boy, diagnosed as oblique ulnar fracture AMF Bado type I, treated with elastic intramedullary nail, after 37 months FU, the movement of elbow return normal

In this study, the EIN Group had shorter operation time and less blood loss than the LCP Group, which may be related to the smaller incision of EIN compared to LCP, and the process of EIN insert is more easier than LCP fixation (Table 2). Both groups showed significant improvements in MEPS, ROM and ROR at three months postoperation and the last follow-up, which suggested that surgical stabilization can provide stable length of ulnar and maintain concentric reduction of radial head for oblique ulnar fracture. Compared to the LCP Group (Fig. 1), there were no significant differences in MEPS, ROM and ROR even in increments of each indicator in the EIN Group, suggesting that EIN provides the same stability to ulna in oblique fracture, both in subjective assessment of rehabilitation and objective clinical evaluation of function restoration (Fig. 2).

**Table 2:** Average results of two groups

Group	Age (yo)	No	FU (m)	OP (min)	Blood Loss (ml)	Pre-OP			3 M Post-OP			LFU Post-OP		
						MEPS	ROM	ROR	MEPS	ROM	ROR	MEPS	ROM	ROR
EIN	7.6 ± 1.3	16	34.4 ± 5.7	48.1 ± 9.2	8.2 ± 2.3	19.4 ± 5.4	16.3 ± 5.6	10.3 ± 3.4	74.7 ± 4.6	79.7 ± 6.7	94.7 ± 7.2	90.3 ± 3.4	135.0 ± 5.8	150.0 ± 8.9
LCP	7.9 ± 1.4	12	32.7 ± 4.2	67.1 ± 7.2	18.8 ± 3.8	19.6 ± 4.0	15.0 ± 3.7	12.1 ± 5.0	74.2 ± 4.7	76.3 ± 5.7	90.8 ± 7.6	88.8 ± 3.1	133.3 ± 4.4	147.9 ± 7.9

Ramski indicated that EIN is recommended for length stable (eg, transverse or short oblique) ulnar fractures and LCP is recommended for length unstable (eg, long oblique or comminuted) ulnar fractures, and fracture pattern was deemed “long oblique” if the fracture line measured > 2 times the cortical diameter [8]. But in this study, it is showed that even in long oblique ulnar fracture case (Fig. 2), if there can formed impact between both end of fracture when reduce, and comfirmed there is no ulnar axial displacement after ulna reduced, choosing proper diameter EIN (more than one half ulnar diameter) could also achieve good clinical result.

This study had some limitations. First, the number of patients enrolled in this study was limited. Second, the follow-up time was insufficient to provide long-term results. Moreover, EIN methods have some disadvantages. For example, LCP can provide more stability than EIN for comminuted ulnar fracture.

## Conclusion

Oblique ulnar Bado type I acute Monteggia fracture in pediatric patients fixed by EIN and LCP can achieve good mid-term results measured by MEPS, ROM and ROR, EIN procedure has shorter operation time and lessbloodloss than LCP procedure.

## Data Availability

The datasets generated and/or analysed during the current study are not publicly available due to privacy of the patients but are available from the corresponding author on reasonable request.

## Abbreviations

EIN: Elastic intramedullary nail
LCP: Locking compression plate
MEPS: Mayo Elbow Performance Score
ROM: Range of motion
F/E: Flexion/Extension
P/S: Pronation /Supination
N/A: Not Applicable
AMF: Acute monteggia fracture
